# RBF-TSS: Identification of Transcription Start Site in Human Using Radial Basis Functions Network and Oligonucleotide Positional Frequencies

**DOI:** 10.1371/journal.pone.0004878

**Published:** 2009-03-16

**Authors:** Rami N. Mahdi, Eric C. Rouchka

**Affiliations:** Department of Computer Engineering and Computer Science, University of Louisville, Louisville, Kentucky, United States of America; Institute of Infectious Disease and Molecular Medicine, South Africa

## Abstract

Accurate identification of promoter regions and transcription start sites (TSS) in genomic DNA allows for a more complete understanding of the structure of genes and gene regulation within a given genome. Many recently published methods have achieved high identification accuracy of TSS. However, models providing more accurate modeling of promoters and TSS are needed. A novel identification method for identifying transcription start sites that improves the accuracy of TSS recognition for recently published methods is proposed. This method incorporates a metric feature based on oligonucleotide positional frequencies, taking into account the nature of promoters. A radial basis function neural network for identifying transcription start sites (RBF-TSS) is proposed and employed as a classification algorithm. Using non-overlapping chunks (windows) of size 50 and 500 on the human genome, the proposed method achieves an area under the Receiver Operator Characteristic curve (auROC) of 94.75% and 95.08% respectively, providing increased performance over existing TSS prediction methods.

## Introduction

The accurate identification of promoter regions and transcription start sites (TSSs) is an important step for in-silico gene discovery and understanding of the transcription regulation mechanisms. Every eukaryotic gene has a core promoter region in the 5′ untranslated region (UTR) that contains at a minimum a TSS signal. Most eukaryotic genes are transcribed by RNA Polymerase 2 (Pol-II) which binds at the TSS. Promoter regions are found to share common subtle patterns or models known as motifs that act as binding sites where other transcription factors (TFs) attach to facilitate or regulate transcription. For example, up to 80% of human promoters contain an initiator element (Inr) located at the transcription start site with a consensus sequence of YCAYYYYY, where Y represents a pyrimidine base C or T [1]. Roughly 30% of human core promoters are found to contain a TATA box at position of −20 to −30 from the TSS with the consensus TATAAA [1]. The TATA box tends to be surrounded by GC rich sequences. Promoter signals with greater variation are found in the promoter region proximal to the TSS, where motifs such as the CAAT, GC, E, and GATA boxes are located [2]. More details about compositional characterization of known human promoter motifs can be found in [2].

### Promoter detection algorithms

A number of algorithms for promoter and TSS recognition are currently available. Each attempts to model promoter pattern(s) using features such as CpG islands and known transcription factor binding sites (TFBS) to distinguish promoters from non-promoters. Some methods such as Autogene [3] and Promoter Scan [4] use position weight matrices (PWM) to signal the presence of a high density of binding sites indicating potential promoters. However, it has been shown that both the location and combination of different binding sites are important for promoter recognition [5], [6]. Eponine [7] improves recognition by associating every PWM with a probability distribution based on its position relative to the TSS. A more recent tool that tries to model the oligonucleotide positional densities is described in [8]. However, this particular design employs a naïve Bayes classifier that assumes every oligonucleotide's positional distribution is independent, and is therefore unable to capture the co-occurrence of a specific combination of binding sites.

In a recent study, Bajic and colleagues conducted a large scale comparison study of eight known TSFs [9]. They demonstrate that a number of these tools perform well, yet leave a lot of room for improving detection accuracy. Among the most successful tools identified were Eponine [7], McPromoter [10], FirstEF [11] and DragonGSF[12].

A more successful approach is the ARTS tool developed by Sonnenburg and colleagues [13] which uses a support vector machine (SVM) with multiple advanced sequence kernels. ARTS is able to achieve a high accuracy with the area under the ROC curve of 92.77% and 93.44% for genomic DNA chunk sizes of 50 and 500 respectively, demonstrating a superiority to Eponine [7], McPromoter [10] and FirstEF [11]. As part of the ARTS system, a large training and testing data set was constructed along with measures for testing and evaluating promoter detection approaches in a consistent fashion. This data set and methodologies are used to compare the results of our approach, RBF-TSS, to ARTS, which has been shown to be the best performing approach previously available. In the comparison section, the performance measures of ARTS, Eponine, McPromoter and FirstEF are listed as they were reported in [13]. Furthermore, we evaluated the performance of a more recent tool, ProStar [14]. ProStar is developed based on a hypothesis that core and proximal regions characterize unique deformation and stiffness properties. From the analysis of the helical stiffness along the human genome, distinctive structural properties were shown to have a strong correlation with annotated TSSs. For a given sequence, ProStar computes a six-dimensional deformation vector v (twist, tilt, roll, shift, slide, rise) for the whole sequence and uses Mahalanobis distance to find the closest class. Due to the limited flexibility of the available ProStar software, it was evaluated at limited number of thresholds and thus the complete ROC and PRC curves were not generated.

### RBF-TSS

We propose a new method to model the positional frequency of oligonucleotides to form a single feature to represent the given sequences for promoter detection. Unlike [8], which measures the frequency at every single base pair position from the TSS, our approach takes the sequence around the TSS and divides it into overlapping windows for which the frequency of oligonucleotides of specific length are measured. A number of different combinations of window sizes, varying overlapping lengths and oligonucleotides length were examined. The combination resulting in the largest area under the ROC curve in classifying the validation data was chosen for the testing phase.

The extracted positional frequency feature is used as an input into RBF-TSS, a classification algorithm for transcription start sites based on a radial basis function neural network (RBFNN). For training, gradient descent learning is used to simultaneously estimate the RBFNN optimal weights, sub-models' centers and sub-models' covariance matrices [15]. RBF-TSS employs a recently published clustering algorithm for initialization that utilizes the large number of available background samples found within genomic DNA [16]. Weight decay is implemented to regularize the classifier [17] and the improved Rprop algorithm (iRprop+) [18] is used for estimating the learning rate factors for the gradient descent learning of the optimal parameters of the network. iRprop+ was shown to be fast, easy to implement and suitable when estimating many different variables since it uses separate learning rate factors for every variable.

The same experiment setting published to test the ARTS method and others in [13] is used to evaluate RBF-TSS. The proposed method showed to be superior to the ARTS in terms of the area under the ROC curve but not in terms of the area under precision recall curve (PRC). However, the PRC might not be a suitable measure of the performance of promoter identification tools since some samples labeled as true negatives might indeed be novel promoter regions. For example, removal of a 100 negative samples out a million causes the area under the PRC to increase by 6.36 and 10.86 with chunk sizes of 50 and 500, respectively, while the area under the ROC remains nearly identical.

## Methods

### Feature Prototype (Local Oligonucleotide Frequencies)

Promoter regions function as such due to the co-occurrence of a specific set of motifs at specific yet flexible distances from the TSS [5], [6]. However, none of the published studies or tools has found a single common pattern that can explain all promoters, indicating the likelihood of multiple promoter patterns.

**Figure 1:**
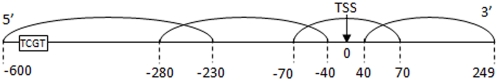
Training sequences are divided around the TSS with overlapping regions. This specific subdivision shows feature 7 settings, as described in Table 1.

In order to capture the characteristics of the given promoter sequences, training sequences with known TSS are divided into overlapping regions (Fig. 1). Either 4-mer or 3-mer oligonucleotide frequencies are measured in every sub-region. All of these sub-frequencies are combined to form a feature vector to describe and represent the given sequence sample. This approach is a compromise between methods that use the frequencies of all oligonucleotides around the TSS regardless of their positions, and those that measure positional densities at every single base relative to the TSS. Knowing the region in which each oligonucleotide occurs yields approximate positional information about the motifs.

Eight combinations of region lengths and overlap sizes are tested to extract separate features, including seven with oligonucleotide of length four and one with oligonucleotide of length three. The overlapping regions considered for each of these combinations are listed in Table 1, with the position relative to the known TSS. These regions are further illustrated in (Fig. 1) for combination seven. In general, regions and overlap areas close to the TSS are short and increase in length as they go farther from the TSS. This is due to the knowledge that common motifs in the core promoter region (close to the TSS) are found to have more strict positions than common motifs found in the promoter proximal region area (farther from the TSS) [5], [6]. After each combination is considered, the one resulting in a classifier with the highest area under the ROC for the validation data is selected for testing.

**Table 1:** Sub-regions and oligonucleotide lengths considered for feature extraction.

Feature	Sub-region ranges (relative to the TSS)	Oligonucleotide
1	(−500,−230),(−300,−50),(−100,20),(−20,99)	4 mer
2	(−500,−220),(−310,−40),(−110,30),(−30,99)	
3	(−500,−240),(−290,−60),(−90,10),(−10,99)	
4	(−600,−230),(−280,−40),(−70,70),(40,199)	
5	(−600,−240),(−270,−50),(−60,60),(50,199)	
6	(−600,−280),(−330,−110),(−150,20),(−20,149)	
7	(−600,−230),(−280,−40),(−70,70),(40,249)	
8	(−650,−490),(−550,−400),(−450,−310),(−350,−220),(−260,−140),(−170,−60),(−90,10),(10,70), (50,150),(120,229)	3 mer

### Radial basis function (RBF)

**Figure 2:**
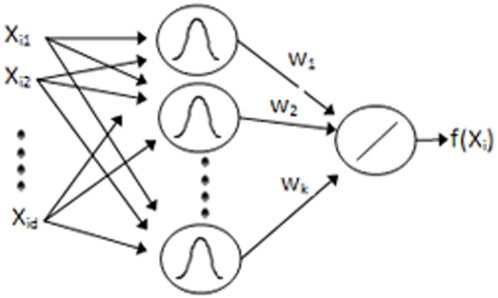
Typical Radial Basis Function network topology.

For learning and classification, a modified version of the radial basis function network trained by three learning phases is employed [15]. Radial basis functions [19] are composed of three layers: input, hidden and output (Fig. 2). The hidden layer nodes use radial basis functions such as a Gaussian membership while the output layer is a weighted sum of the outputs from the hidden layer nodes. Such a network has been shown to be useful in classification problems [20].

Typically, training radial basis function neural networks is performed in two phases. First, the parameters (means and variances) of the hidden layers nodes are estimated using clustering or a density estimation algorithm. Afterward, either gradient descent or a pseudo inverse solution is used to estimate the optimal weights to minimize the error criterion described in Eqn. (1) Where 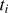 is assigned the value of 0 or 1 depending on the true label of training sample 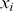 and 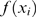 is the output score computed by the network using Eqn. (2).
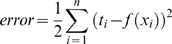
(1)

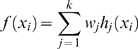
(2)


In Eqn. (2) 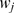 is the weight assigned to node (j) in the hidden layer and 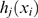 is the membership of sample 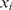 to node (j). In the case of the Gaussian function being used to compute the membership, 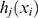 is computed using Eqn. (3).
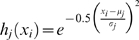
(3)


It has been shown that using a third phase learning process where the means, variances and the weight are all being estimated simultaneously by gradient descent and back propagation provides significant improvement on classification accuracy [15].

We improve on the three phase learning RBF in three different ways. First, instead of initializing the hidden layer nodes using the K-means clustering algorithm, we initialize them using a modified k-means algorithm accompanied by split and merge operations [16] where abundant background samples are used to estimate the number of clusters while at the same time avoiding non-descriptive local minimums. Clustering is performed among promoter samples only while non-promoter samples are considered background. Split and merge operations are performed in a direction to minimize the overlap between the clusters of promoters and background samples.

The second improvement is derived from observations in neural networks where it has been demonstrated that keeping weights at low variation and small values increases the performance of classifier [17]. This is accomplished by adding a new term to the objective function in Eqn. (1) that penalizes the large weight values using Eqn. (4).
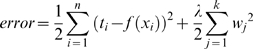
(4)


In Eqn. (4), 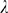 is a constant determined by validation data. After adding the new term and using gradient descent, it can be shown that in order the minimize the error value for Eqn. (4) the weights, means and variances need to be iteratively updated as given in Eqns. (5–7), respectively.
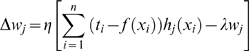
(5)

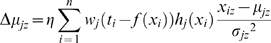
(6)

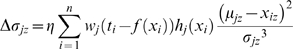
(7)


In Eqns. (5–7), 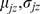 are the mean and the standard deviation of node j along dimension z while 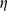 is an estimated learning rate factor.

The third improvement over the three phase RBF learning algorithm is to replace the common learning rate 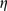 factor by separate factors for every variable and use the improved RProp algorithm (iRprop+) [18] to estimate learning factors at every iteration. The iRprop+ has been shown to increase the speed of learning by adaptively estimating separate learning rates for every variable. This last step is critical since there are different variables that are scaled differently and hence are very likely to demand changes with different rates. Furthermore, the iRprop+ algorithm employs a backtracking mechanism where updates that worsen the learning are rescinded.

### Training and model selection

Eight different features were extracted as described in the “Feature Prototype” section. Each dimension was normalized by dividing by the mean. Clustering to initialize the RBF network was performed using k-means with split and merge operations as described in [16]. We used the default settings with merge and split thresholds of 0.5 and 0.9 respectively while the clustering misclassification of the background samples was weighted by 0.5. The large thresholds and the weighting correspond to accepting relatively high noise to address the abundance of background samples and to avoid a large number of clusters.

Initially, for every feature, a separate RBF network was constructed without weight regularization (λ = 0). The two best performing features in classifying validation data were chosen for further training. Those two features were four and seven (Table 1). Both features were extracted by measuring the frequency 4-mers in four overlapping sub-regions of the given sequences as described in Table 1. Afterward, different values for λ (0.5, 1, 2, 4, 8, 16, 32, 64 and 256) to regularize the weights were tried with both features seven and four separately. Feature seven with λ = 64 was found to generate the best performance with an area under the ROC curve of 93.58% for validation data and 96.7% for training data.

**Figure 3:**
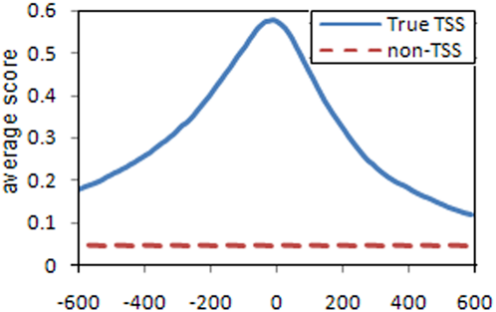
Average scores at positions around the true TSS vs. average scores of negative examples in validation data. The x-axis represents the relative position to the true TSS within the positive examples.

Figure 3 shows the average single base validation data score of the final network in the range [−600 to 600] around the known TSS position compared to the average score for negative examples. At every base, the feature vector was extracted using sub-regions as if that base was the TSS. It is clear from the curve that the classifier is able to produce output scores capable of distinguishing positive from negative examples. These scores get significantly higher the closer we get to the true TSS.

Given the high dimensionality of the proposed feature prototype and the different choices, building the proposed model with the different parameters for model selection demanded heavy computation. The approximate time needed to build a whole model for a single feature choice varied. Using a 3.0 GHz Xeon processor, a single clustering demanded 8–14 hours while training the un-regularized RBF network took 7–9 hours and the time needed for convergence increased as the regularization parameter increased up to 3× with λ = 64. On the other hand, a memory of 2GB was quite enough for a single process. However, with the availability of multi-core processors, we were able to cut the training time significantly as we tried different configurations on different cores.

## Results

### Data set

The data set used for evaluating ARTS [13] was downloaded from (http://www.fml.tuebingen.mpg.de/raetsch/projects/arts) and used to evaluate RBF-TSS. This data set is divided into three parts: training, validation and testing. As a summary of the ARTS paper, the training and validation were extracted from the dbTSS version 4 (dbTSSv4) [21] which is based on the UCSC human genome sequence assembly and annotation version 16 (“hg16”) [22]. RefSeq [23] identifiers from dbTSSv4 were used to extract the corresponding mRNA using NCBI nucleotide batch retrieval. Afterward, they aligned all the retrieved mRNA from NCBI to hg16 genome using BLAT [24]. The best alignment position at the genome was compared to the putative TSS positions as stated in dbTSSv4. Sequences whose positions did not meet the following checks were discarded: 1.Chromosome and strand of the TSS position and of the best BLAT hit match. 2. The TSS position is within 100 base pairs from the gene start as found by the BLAT alignment. 3. There does not exist a processed putative TSS within 100 bp of the current one. A total of 8,508 genes were accepted and positive examples were extracted as a window of size [−1200, +1200] around the TSS.

For this dataset, 85,042 negative samples were created by randomly extracting 10 subsequences of window length [−1200, +1200] from the interior of every gene between 100 bp downstream of the known TSS and the end of the gene [9]. This method is arguable since it cannot be guaranteed these negative samples do not contain promoters. However, it is near certain most of the extracted negative samples are true negatives since TSS are found to be rare compared to the size of the genome. Furthermore, there is not any other natural method of recognizing true negatives in the genome.

The 8,508 positive and 85,042 negatives examples were both divided into 50% for training and 50% for validation. The testing data set was extracted as the set of all new genes from dbTSSv5 [25] which is based on hg17 and did not appear in dbTSSv4. Genes that have more than a 30% mRNA overlap are removed from consideration.

### Testing procedure

We performed the same testing procedure as described in [13]. Every chromosome strand was divided into non-overlapping chunks of size 50 and 500 bases. Any chunk that falls within 20 bp from any known TSS position of any of the testing genes was considered as a positive sample. Any chunk that falls between +20 bp downstream of the start of any of these genes to the end of the same gene and was not labeled positive was considered a negative sample. In the case of chunks of size 50, the number of positive and negative examples were 1,588 and 1,087,666 respectively while in the case of chunks of size 500 they were 943 and 108,782 respectively. Non- ACGT bases (i.e. long runs of N's) were randomly substituted by A, T, C or G.

For every chunk, a feature vector was extracted at every single base as if that base was the TSS position. A network score is computed at every base and each chunk is assigned the maximum value found for any of the bases contained within it. This may result in chunking and labeling of positive samples despite being up to 20 bp away from the true TSS. This is by design to acknowledge the flexibility of POL-II which does not always bind to a specific single base but rather anywhere in the range [−20, +20] from the start of the TSS. The difference in distribution of scores calculated by RBF-TSS for true TSS and non-TSS sequences is shown in (Fig. 3).

**Figure 4:**
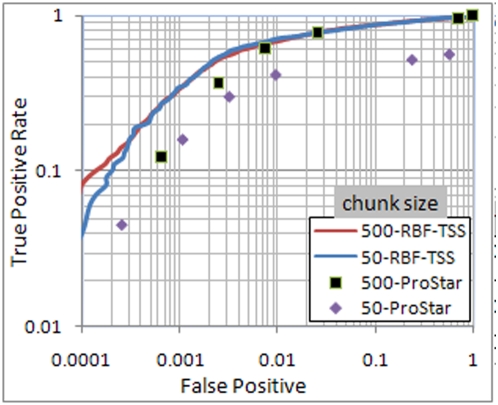
ROC curve for chunk sizes 50 and 500. Both axes are scaled to logarithm base 10 to highlight the difference.

**Figure 5:**
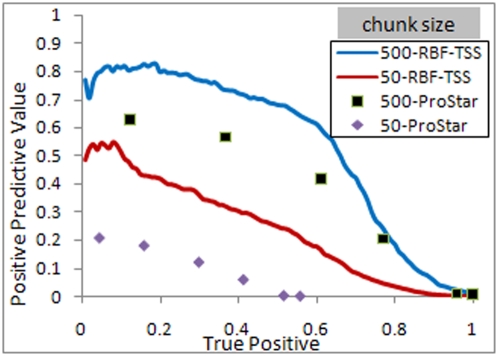
PRC curve for chunk sizes of 50 and 500.

The true positive rate (TPR) for TSS identification was calculated as the percentage of positive samples identified as such by RBF-TSS while the false positive rate was calculated as the percentage of true negative samples mistakenly labeled as positive. A comparison of these rates is shown in Figs. 4 and 5. The positive predictive value (PPV) is calculated as the ratio of the positive samples whose true label is positive to the total number of samples classified as positive. As illustrated in Fig. 5, the area under the precision recall curve is relatively low due to the fact that the ratio of negative to positive samples is very high, and varies widely between the two cases of chunk size of 50 and 500.

### Comparison to Other Methods

The performance of RBF-TSS was compared to other methods using both the area under the ROC and PRC curves (Table 2). Note that the results for the ARTS, Eponine, McPromoter and FirstEF methods are taken as reported in [13]. As seen in Table 2, the proposed method has better performance in terms of area under the ROC curve in both chunk size cases 50 and 500. Furthermore, the similar performance between chunks of size 50 and 500 indicates high locality of the proposed method for locating the TSS positions as compared to the other methods.

**Table 2:** Area under the curve for RBF-TSS and ARTS.

Curve	auROC %		auPRC %	
Chunk Size	50	500	50	500
RBF-TSS	94.75	95.08	24.08	54.64
ARTS	92.77	93.44	26.18	57.19
Eponine	88.48	91.51	11.79	40.80
McPromoter	92.55	93.59	6.32	24.23
FirstEF	71.29	90.25	6.54	40.89

On the other hand, the proposed method fails to exceed the ARTS method when using area under precision recall curve. This should be of no surprise since it has been analytically shown in [26] that optimizing the area under the ROC curve is not guaranteed to optimize the area under the PRC curve.

The precision recall curve is found to be very sensitive to having few negative samples with high scores with RBF-TSS. For example, the removal of the 100 negative samples with the highest network scores results in a change of the auPRC from 24.08% to 30.44% and 54.64% to 65.5% for chunk sizes of 50 and 500 respectively. In contrast, the change in the auROC was minimal, increasing from 94.75% to 94.76% and 95.08% to 95.14% for chunk sizes of 50 and 500 respectively. These 100 samples represent less than 0.1% of the negative samples, yet their removal illustrates the sensitivity of the PRC. The use of the auPRC should be considered with caution as an evaluation measure of TSS finders since the PRC has a demonstrated sensitivity. Mislabeled negative samples could be unknown TSS, which is shown to potentially have a significant effect on the auPRC.

## Discussion

A new novel feature is proposed that transforms the problem from sequences and temporal space to Euclidian space. Such a feature makes it possible to cluster promoter sequences and build an RBF neural network.

A key advantage of the proposed method is that once training the RBF network is finished, the set of resulting neurons with positive weights can be perceived as a mixture of Gaussians representing promoter samples probability distribution in the new Euclidean space. Such knowledge can pave the way for a higher level analysis in the time space. For example, having many promoter sequences with high membership to one neuron indicates that they belong to one cluster and hence share many of their of oligonucleotides' frequencies in the same sub-regions. Therefore, it becomes more efficient to restrict motif searching or multiple alignment to this set of promoters.

The proposed RBF-TSS method has demonstrated high accuracy performance in detecting transcription start sites and proven to be very competitive to the high performing ARTS tool and others. The proposed method achieved area under the ROC of 94.75% and 95.08% for chunks of size 50 and 500 as compared to 92.77% and 93.44% achieved by the ARTS using the same data set and testing procedure. The high performance of the proposed method with chunk size of 50 proves that RBF-TSS has increased the classification accuracy over previously described TSS prediction algorithms, and performs well with high locality precision.

An executable Java JAR file for RBF-TSS is available for free download at: http://bioinformatics.louisville.edu/RBF-TSS/. This website contains additional supporting materials, including training and testing data and a more detailed description of the RBF-TSS algorithm.
